# Periodontal condition and ultrasound-based measures of arterial stiffness: results of the Health 2000 Survey

**DOI:** 10.1186/s12903-022-02502-w

**Published:** 2022-11-12

**Authors:** Emilia Ollikainen, Tellervo Tervonen, Anna Liisa Suominen, Matti Knuuttila, Antti Jula, Tuomas Saxlin, Pekka Ylöstalo

**Affiliations:** 1 Research Unit of Oral Health Sciences, Faculty of Medicine, University of Oulu, PO Box 5000, 90014 Oulu, Finland; 2 Medical Research Center Oulu, Oulu University Hospital and University of Oulu, Oulu, Finland; 3 Institute of Dentistry, University of Eastern Finland, Kuopio, Finland; 4 Oral Health Teaching Clinic, Kuopio University Hospital, Kuopio, Finland; 5 Department of Public Health and Welfare, Finnish Institute for Health and Welfare (THL), Kuopio, Finland; 6 Finnish Institute for Health and Welfare (THL), Turku, Finland

**Keywords:** Arterial stiffness, Gingival bleeding, Periodontal pocket, Ultrasound

## Abstract

**Background:**

Periodontitis has been associated with inflammatory processes in arterial walls such as impairment in endothelial function and thickening of intima media. As inflammation plays a role also in arterial stiffening, an association between periodontal inflammation and arterial stiffness can be expected. So far, conflicting results of the association between periodontal disease and arterial stiffness have been reported. Many of the earlier studies were conducted in specific populations and heterogeneous measures of both arterial stiffness and periodontal status were used. In this population-based study we aimed to investigate whether periodontal pocketing and gingival bleeding are associated with ultrasound-based measures of arterial stiffness.

**Methods:**

In this cross-sectional study, two sets of data based on the national Health 2000 Survey in Finland were formed. Data set I comprised never-smoking 45–64-year-old dentate (≥ 10 natural teeth), non-diabetic, non-rheumatic, non-obese (BMI ≤ 30 kg/m^2^), non-hypertensive subjects with no coronary artery disease or ongoing lipid-lowering medications (n = 157). Data set II was formed of an unrestricted 45–74-year-old dentate population (n = 536). Four arterial stiffness measures (carotid artery compliance, Peterson’s elastic modulus, Young’s elastic modulus and beta stiffness index) based on an ultrasound examination of the common carotid artery were used. Periodontal parameters included the number of teeth with ≥ 4 mm deep periodontal pockets and the number of sextants with gingival bleeding. β-estimates, confidence intervals, and *p*-values were obtained from linear regression models.

**Results:**

In Data set I, the adjusted β-estimates for the association between the number of teeth with ≥ 4 mm deep periodontal pockets and Peterson’s elastic modulus and Young’s elastic modulus were 15.80 (*p* = 0.12) and 61.02 (*p* = 0.22), respectively. The respective β-estimates were 31.06 (*p* = 0.17) and 121.16 (*p* = 0.28) for the association between the number of bleeding sextants and these two stiffness measures. The results in Data set II were in line with the results in Data set I, with the exception that the adjusted β-estimates for the associations between Peterson’s elastic modulus and Young’s elastic modulus and periodontal parameters were closer to null.

**Conclusions:**

This population-based study did not provide evidence of an association between periodontal condition and arterial stiffness.

## Background

The term “arterial stiffness” is used to indicate decreased elastic vessel wall properties as a consequence of degenerative changes in the extracellular matrix of the intima media layer. The most important changes are elastin fragmentation, collagen deposition, the crosslinking of collagen and elastin fibers by advanced glycation end-products, and vessel wall calcification. The principal determinants for arterial stiffness are age and blood pressure while, according to various studies, many of the traditional risk factors for cardiovascular diseases, such as smoking, obesity, dysglycemia and dyslipidemia, play a minor [1–3] or even an insignificant [4] role in arterial stiffening. The current evidence also shows that arterial stiffness is a predictor of future cardiovascular disease [3, 5, 6].

Atherosclerosis, an inflammatory condition of the intima layer of the blood vessel wall, is characterized by lipid accumulation, influx of inflammatory cells, vascular smooth muscle cell migration and development of foam cells. A large number of studies have shown associations between periodontitis and atherosclerotic/cardiovascular diseases [7]. Importantly, the two pathologic processes, atherosclerosis and arterial stiffening, share common pathophysiologic mechanisms, interact in a complex manner and may potentiate each other [3].

Common periodontal diseases, gingivitis and periodontitis, have been linked with low-grade systemic inflammation [8, 9]. Besides the inflammatory effects of periodontal diseases on a systemic level, periodontal pathogens are capable of inducing inflammatory responses locally in the arterial walls and even invade the endothelial cells [10, 11]. In the light of the fact that inflammation seems to be a crucial factor and a major contributor also in arterial stiffening [12], an association between periodontal diseases and arterial stiffness can be hypothesized.

So far, the association of periodontal condition with arterial stiffness has been examined in a number of studies [13–16]. Although there is some support for an association, the credibility of the findings of the earlier studies can be questioned. The reason is that many of the studies were conducted in specific populations, for example in patients with cardiovascular diseases or type 2 diabetes or in individuals seeking dental/medical care. In addition, heterogeneous measures of both arterial stiffness and periodontal status were used.

In this population-based study, we used the data of the national Health 2000 Survey in Finland to investigate the possible link between periodontal condition and peripheral vascular function. Our aim was to study whether clinical periodontal parameters—pocketing and gingival bleeding—are associated with ultrasound-based arterial stiffness measures, such as carotid artery compliance, Peterson’s elastic modulus, Young’s elastic modulus and beta stiffness index.

## Methods

The national Health 2000 Survey was carried out in Finland by the Finnish Institute of Health and Welfare (THL). The data were collected using clinical health and oral examinations, laboratory analyses, self-administered questionnaires and personal interviews. This study is a secondary analysis of the data of the Health 2000 Survey and its complementary investigation of the circulatory system, which was conducted about 1 year after the actual Health 2000 Survey.

The data on arterial stiffness were from the complementary investigation of the circulatory system. In all, 1,867 individuals, aged between 45 and 74 and living in the catchment areas of the five Finnish university hospitals, were invited to the complementary investigation, and of those 1,526 (82%) agreed to participate.

### Inclusion criteria

Data sets I and II were formed as follows of those subjects who participated both in the complementary health examination and in the actual Health 2000 Survey, in which periodontal data were collected.

### Data set I

**Fig. 1:**
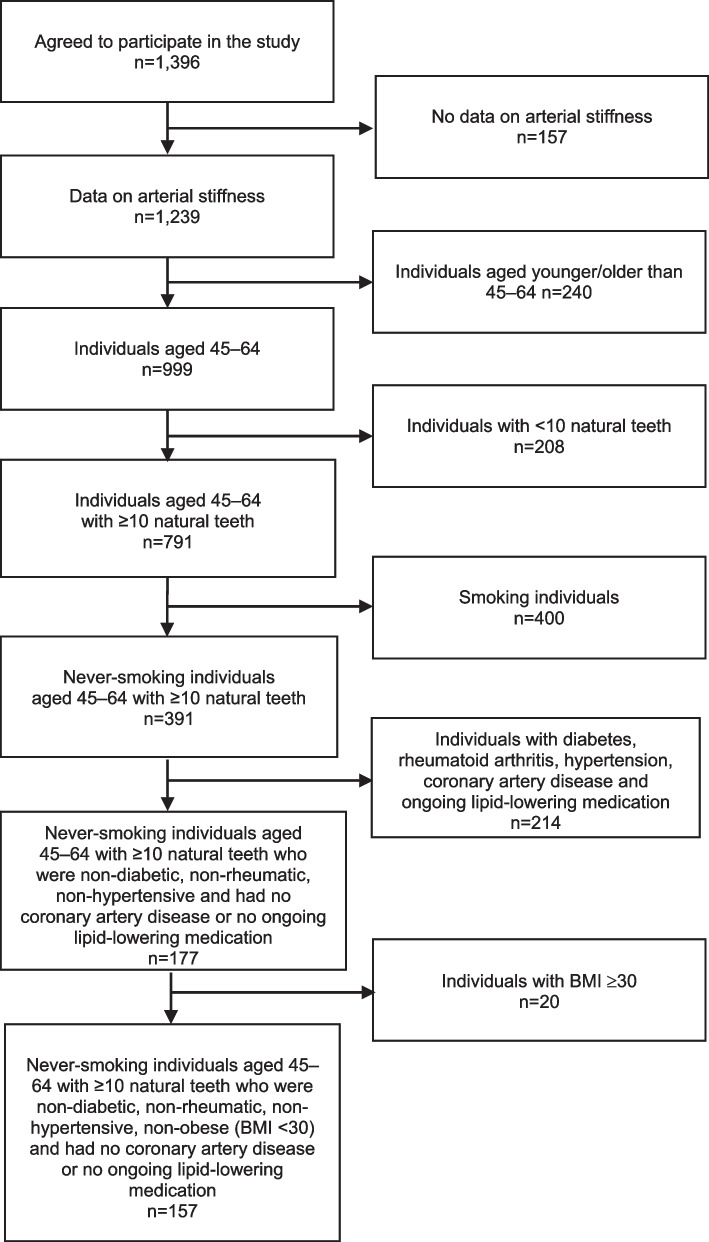
Formation of the study population in Data set I

Data set I comprised never-smoking, dentate (≥ 10 natural teeth), non-obese (body mass index [BMI] ≤ 30 kg/m^2^), non-diabetic, non-rheumatic and non-hypertensive individuals aged 45–64, with no coronary artery disease or ongoing lipid-lowering medications (n = 157) (Table 1, Fig. 1).

**Table 1:** Basic characteristics of the study population presented in the total population and in the different categories by the number of teeth with ≥ 4 mm deep periodontal pockets (Data set I, n = 157)

	Number of teeth with ≥ 4 mm deep periodontal pockets					
	Total	0	1–3	4–6	7–11	12 +
	(n = 157)	(n = 52)	(n = 43)	(n = 23)	(n = 28)	(n = 11)
Age (mean)	51.8	50.8	51.7	54.0	52.2	51.6
Gender, proportion of males (%)	27.6	23.2	21.2	42.9	29.4	45.5
*Educational level (%)*						
Low	24.7	19.6	28.9	23.8	29.4	18.2
Intermediate	29.3	30.4	38.5	23.8	17.7	27.3
High	46.0	50.0	32.7	52.4	52.9	54.6
*Serum lipid composition mmol/l (mean)*						
Total cholesterol	5.9	5.6	6.1	6.0	5.9	6.1
Triglycerides	1.2	1.2	1.1	1.2	1.1	1.6
LDL-cholesterol	3.6	3.3	3.8	3.7	3.6	4.0
HDL-cholesterol	1.5	1.6	1.5	1.4	1.6	1.3
*BMI (mean)*	24.2	23.8	24.4	24.4	24.5	24.7
*Level of physical activity (%)*						
Ideal	10.9	12.5	9.6	19.1	8.8	0
Sufficient	33.9	30.4	42.3	23.8	35.3	27.3
Intermediate	27.6	28.6	25.0	33.3	29.4	18.2
Sedentary	27.6	28.6	23.1	23.8	26.5	54.6
*Number of teeth*						
Mean	25.3	24.4	26.1	26.0	25.3	27.4
Median	27.0	26.0	26.0	27.0	27.0	28.0
*Number of teeth with* ≥ *4 mm deep periodontal pockets*						
Mean	3.8	0.0	2.0	4.9	8.6	15.0
Median	2.0	0.0	2.0	5.0	8.0	15.0
*Number of bleeding sextants*						
Mean	2.8	1.9	2.2	3.0	4.3	4.7
Median	3.0	1.5	2.0	3.0	4.0	6.0
*Systolic blood pressure mmHg*						
Mean	122.0	119.3	123.6	123.5	122.4	124.2
Median	121.3	119.7	121.7	121.7	119.5	121.3
*Diastolic blood pressure mmHg*						
Mean	74.3	73.9	75.3	74.0	74.2	73.3
Median	74.3	74.5	75.7	69.0	73.7	73.0
*Pulse pressure mmHg*						
Mean	47.7	45.4	48.3	50.0	48.2	50.9
Median	47.0	46.2	46.0	49.0	47.0	47.3
*Peterson’s elastic modulus mmHg*						
Mean	1044.1	967.8	1103.0	947.2	1053.0	1355.0
Median	912.0	840.5	927.0	859.0	960.0	863.0
*Young’s elastic modulus mmHg*						
Mean	5159.1	4853.7	5478.2	4548.5	5114.5	6746.5
Median	4183.0	3988.5	4593.0	3991.0	4166.0	5112.0
*Carotid artery compliance %/10 mmHg*						
Mean	1.2	1.3	1.1	1.3	1.1	1.2
Median	1.1	1.2	1.1	1.2	1.0	1.2
*Beta stiffness index*						
Mean	3.5	3.4	3.5	3.4	3.5	3.6
Median	3.4	3.4	3.5	3.3	3.5	3.2

### Data set II

**Fig. 2:**
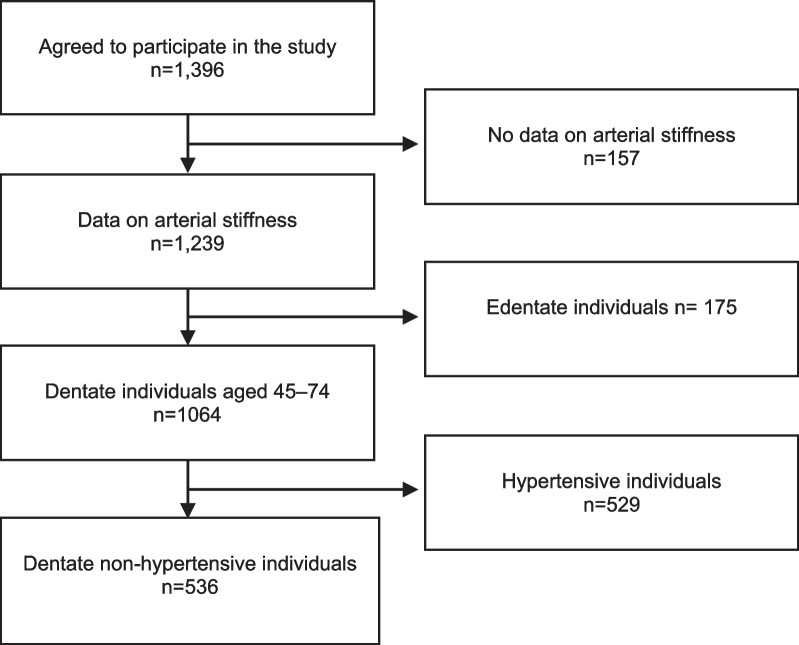
Formation of the study population in Data set II

Data set II consisted of non-hypertensive, dentate (≥ 1 natural tooth) individuals aged 45–74 (n = 536) (Table 2, Fig. 2).

**Table 2:** Basic characteristics of the study population presented in the total population and in the different categories by the number of teeth with ≥ 4 mm deep periodontal pockets (Data set II, n = 526)

	Number of teeth with ≥ 4 mm deep periodontal pockets					
	Total	0	1–3	4–6	7–11	12 +
	(n = 526)	(n = 148)	(n = 145)	(n = 90)	(n = 82)	(n = 71)
Age (mean)	53.9	53.0	53.6	55.4	54.4	54.2
Gender, proportion of males (%)	41.4	29.1	37.9	50.0	42.7	62.0
*Educational level (%)*						
Low	29.9	27.7	30.3	27.8	31.7	33.8
Intermediate	32.7	33.8	34.5	33.3	29.3	29.6
High	37.5	38.5	35.2	38.9	39.0	36.2
*Serum lipid composition mmol/l (mean)*						
Total cholesterol	6.0	5.9	6.0	5.8	6.1	6.2
Triglycerides	1.3	1.2	1.2	1.1	1.2	1.3
LDL-cholesterol	3.7	3.5	3.7	3.5	3.8	3.8
HDL-cholesterol	1.4	1.5	1.4	1.4	1.4	1.3
*BMI (mean)*	25.6	24.4	25.4	25.0	25.9	25.3
*Level of physical activity (%)*						
Ideal	7.5	8.1	8.3	12.6	4.9	1.4
Sufficient	31.0	27.7	39.3	25.3	30.5	28.2
Intermediate	29.6	29.7	26.9	31.0	36.6	25.4
Sedentary	31.9	34.5	25.5	31.0	28.1	45.1
*Number of teeth*						
Mean	22.8	21.2	22.6	22.9	23.4	25.4
Median	26.0	25.0	25.0	25.5	26.0	26.0
*Number of teeth with* ≥ *4 mm deep periodontal pockets*						
Mean	4.9	0.0	1.8	5.0	8.6	17.1
Median	3.0	0.0	2.0	5.0	8.0	16.0
*Number of bleeding sextants*						
Mean	2.5	1.4	1.9	2.3	1.7	4.8
Median	2.0	1.0	2.0	2.0	4.0	6.0
*Systolic blood pressure mmHg*						
Mean	125.5	123.9	125.7	125.4	125.6	128.6
Median	124.0	123.2	124.0	123.2	124.3	128.0
*Diastolic blood pressure mmHg*						
Mean	75.6	75.7	75.6	74.5	75.3	77.2
Median	75.0	75.2	75.7	74.5	74.8	76.7
*Pulse pressure mmHg*						
Mean	49.9	48.2	50.1	50.9	50.3	51.5
Median	48.5	47.3	47.7	49.5	48.2	50.7
*Peterson’s elastic modulus mmHg*						
Mean	1151.6	1161.7	1092.5	1212.3	1130.5	1198.4
Median	949.0	935.0	927.0	964.0	951.5	963.0
*Young’s elastic modulus mmHg*						
Mean	5644.4	5677.3	5399.4	5957.1	5479.6	5870.2
Median	4589.5	4430.0	4709.0	4778.5	4497.5	4534.0
*Carotid artery compliance %/10 mmHg*						
Mean	1.1	1.1	1.1	1.1	1.1	1.1
Median	1.1	1.1	1.1	1.0	1.1	1.0
*Beta stiffness index*						
Mean	3.5	3.6	3.5	3.6	3.5	3.5
Median	3.5	3.5	3.5	3.4	3.5	3.5

### Measurement of arterial stiffness

The measurement of arterial stiffness (carotid artery compliance, Peterson’s elastic modulus, Young’s elastic modulus and beta stiffness index), measured in complementary investigations, was based on an ultrasound examination of the common carotid artery according to a standardized protocol using a 7.5 or 10 MHz linear array transducer [17, 18]. The examination was performed by trained and certified sonographers [19].

Carotid artery compliance (CAC) measures the ability of the arteries to expand as a response to pulse pressure caused by cardiac contraction and relaxation and is defined as the absolute volume increase within an arterial segment during the cardiac cycle divided by the arterial pulse pressure: {CAC = [(D_s_ − D_d_)/D_d_]/(P_s_ − P_d_)} [17]. High values indicate high distensibility of arterial walls, i.e. low level of arterial stiffness.

Peterson’s elastic modulus (PEM) is the ratio of stress to strain and is an inverse measure of carotid artery compliance: {[(P_s_ − P_d_) × D_d_]/(D_s_ − D_d_)}. Peterson’s elastic modulus can be interpreted as the pressure change that is required for a theoretical 100% increase in the lumen diameter [20]. High values indicate a high level of arterial stiffness.

Young’s elastic modulus (YEM) gives an estimate of arterial stiffness that is independent of intima media thickness (IMT): {YEM = [(P_s_ − P_d_) × D_d_]/[(D_s_ − D_d_)/IMT]} [17]. High values indicate a high level of arterial stiffness.

The beta stiffness index (BSI) was developed to reduce the curvilinear pressure-stiffness relationship and is considered to be a relatively independent indicator of arterial stiffness: {BSI = ln (P_s_/P_d_)/[(D_s_ − D_d_)/D_d_]} [21]. High values indicate high level of arterial stiffness.

In all the above formulas, D_s_ is the systolic diameter, D_d_ is the diastolic diameter and IMT is the intima media thickness of the right common carotid artery, P_s_ is systolic blood pressure and P_d_ is diastolic blood pressure.

Blood pressure (mmHg) was measured from the right upper arm using an automatic blood pressure monitor Omron M4 (Omron Healthcare Group, Kioto, Japan). The subjects were asked to sit down for at least ten minutes before their blood pressure was measured. The measurement was taken three times at one-minute to two-minute intervals. The average of the three measurements was used in the analysis. Pulse pressure was calculated as the difference between the average systolic and the average diastolic blood pressure.

### Periodontal examination

The clinical oral examinations of the actual Health 2000 Survey were carried out in a dental chair by five calibrated dentists using a headlamp, a mouth mirror, a fiber optic light and a WHO periodontal probe. Probing force (20 g) was calibrated using a letter scale. Periodontal pocket depth was measured in millimeters on four surfaces of each tooth, except the third molars and the radices, in the following order: distobuccal, mid-buccal, mid-oral and mesio-oral. Only the depth of the deepest pocket was recorded for each tooth as follows: no deepened periodontal pocket, periodontal pocket 4–5 mm deep, and periodontal pockets 6 mm deep or deeper. For the analyses, a variable–the number of teeth with ≥ 4 mm deep periodontal pockets–was formed to describe the extent of periodontal pocketing and the variable was used as a continuous variable. The agreement between the field examiners and the reference examiner in measuring periodontal pocket depth was 77% (ҡ 0.41). The intra-examiner reliability assessments of periodontal pocket depth produced a ҡ value of 0.83 [22].

Gingival bleeding was registered immediately after the pocket depth measurements in each sextant. The presence of gingival bleeding was categorized as follows: no bleeding on probing, bleeding on probing in one or two sextants, and bleeding on probing in three or more sextants. The agreement between the field examiners and the reference examiner on the presence of bleeding on probing was 66% (ҡ 0.36). The intra-examiner reliability assessments of the presence of bleeding on probing produced a ҡ-value of 0.66 [22].

### Other variables

Information about age and gender were obtained from the population registers. Information about education, which was obtained from the interview, was classified into three categories: low education, intermediate education and high education. The lowest category included participants whose education was below high school level or who had no formal vocational qualifications. The intermediate category comprised those who had graduated from a high school or a vocational school. Furthermore, the highest category included participants who had graduated from a university or from a university of applied sciences [19].

Information about diabetes mellitus was obtained from the health interview and laboratory measurements; the participants were classified as non-diabetics if they had not been diagnosed as having diabetes (type 1 or 2) by a physician and their fasting plasma glucose level (Glucose Hexokinase, Olympus System Reagent, Germany) was < 7.0 mmol/l and the plasma glucose level in the standard two-hour oral glucose tolerance test was < 11.1 mmol/l. The participants were classified as non-hypertensive if they had not been diagnosed as having hypertension, they had no anti-hypertensive medication (both based on the health interview) and their blood pressure was < 140/90 mmHg in the clinical examination. Information about coronary artery disease, rheumatoid arthritis and ongoing anti-hypertensive and lipid-lowering medications were obtained from the health interview. The participants were defined as non-rheumatics if they not been diagnosed as having rheumatoid arthritis. The participants were classified as having no coronary artery disease if they had no angina pectoris or any other indications of coronary artery disease.

Serum triglycerides were analyzed enzymatically (Olympus System Reagent, Olympus Life Science Research Europa GmbH, Munich, Germany). High-density lipoprotein (HDL) cholesterol (mmol/l) and low-density lipoprotein (LDL) cholesterol (mmol/l) values were measured using direct methods based on immunocomplex separation followed by an enzymatic cholesterol determination (Roche Diagnostics, Mannheim, Germany). The analyses were performed on an Olympus AU 400 clinical chemistry autoanalyzer (Olympus Diagnostica GmbH, Hamburg, Germany) [23].

Body weight was measured by body mass index (BMI), which is a measure of body weight in relation to height (kg/m^2^). Information about height and weight were obtained primarily from the clinical health examination, and if this was not possible, the self-reported data from the questionnaire were used [19].

Information about physical activity was based on the questionnaire data. Two scales, the Gothenburg’s scale and the International Physical Activity Questionnaire (IPAQ) scale were used to measure the amount of leisure-time activity, housework, walking and sitting, and the level of physical activity was categorized into four categories (ideal, sufficient, intermediate, sedentary) [19].

Information about smoking history was gathered from the health interview and was categorized as daily smokers, occasional smokers, former smokers who had quit smoking during the past year, former smokers who had quit smoking more than a year ago, and never-smokers who had never smoked [19].

Information about alcohol consumption was obtained from a questionnaire, and the estimated amount of alcohol consumption (g/week) was used as a continuous variable [24].

Additional information about the Health 2000 Survey is available in the reports published by Aromaa & Koskinen [24] and by Heistaro [19].

### Statistical methods

Arterial stiffness measures were used as outcome variables and the number of teeth with ≥ 4 mm deep periodontal pockets and the number of bleeding sextants as explanatory variables. Adjusted β-estimates with 95% confidence intervals (CI) and *p*-values were obtained from linear regression models.

In the analyses of Data set I, adjustments were made for age, gender, educational level, serum lipid composition (triglycerides, LDL-cholesterol, HDL-cholesterol), body weight (BMI), alcohol consumption, the level of physical activity and systolic blood pressure.

In the analyses of Data set II, in which the study population also included smokers and individuals with rheumatoid arthritis, diabetes and coronary artery disease, additional adjustments were made for smoking, diabetes, coronary artery disease and rheumatoid arthritis.

Linear regression analyses were performed using the SAS GENMOD procedure with normal distribution and identity link function (version 9.4).

## Results

The basic characteristics of the study population according to the categories of the number of teeth with ≥ 4 mm deep periodontal pockets are presented in Table 1 (Data set I) and Table 2 (Data set II), and the unadjusted β-estimates with their 95% CI and *p*-values for the number of teeth with ≥ 4 mm deep periodontal pockets and the number of bleeding sextants in Table 3.

**Table 3:** Unadjusted associations of the number of teeth with ≥ 4 mm deep periodontal pockets and the number of bleeding sextants with arterial stiffening in Data sets I (n = 157) and II (n = 536)

	Data set I			Data set II		
	β-estimate	95% CI	*p*-value	β-estimate	95%CI	*p*-value
*Number of teeth with* ≥ *4 mm deep periodontal pockets*						
Peterson’s elastic modulus (mmHg)	19.22	− 1.53 to 39.97	0.07	6.38	− 3.11 to 15.86	0.19
Young’s elastic modulus (mmHg)	87.56	− 14.36 to 189.48	0.09	31.69	− 15.76 to 79.14	0.19
Carotid artery compliance (%/10 mmHg)	− 0.01	− 0.03 to 0.01	0.34	− 0.00	− 0.01 to 0.00	0.20
Beta stiffness index	0.01	− 0.01 to 0.02	0.33	0.00	− 0.00 to 0.01	0.52
*Number of bleeding sextants*						
Peterson’s elastic modulus (mmHg)	38.13	− 8.37 to 84.63	0.11	21.80	− 5.28 to 48.90	0.11
Young’s elastic modulus (mmHg)	150.21	− 78.51 to 378.93	0.20	126.38	− 9.07 to 261.82	0.07
Carotid artery compliance (%/10 mmHg)	− 0.02	− 0.06 to 0.01	0.21	− 0.02	− 0.04 to 0.00	0.09
Beta stiffness index	0.03	− 0.00 to 0.06	0.08	0.01	− 0.01 to 0.03	0.30

Unadjusted β-estimates with 95% confidence intervals (CI) and *p*-values

### Data set I

The adjusted β-estimates from the linear regression models for the association between the number of teeth with ≥ 4 mm deep periodontal pockets and Peterson’s elastic modulus and Young’s elastic modulus were 15.80 (*p* = 0.12) and 61.02 (*p* = 0.22), respectively. For the association between the number of bleeding sextants and these two stiffness measures the respective β-estimates were 31.06 (*p* = 0.17) and 121.16 (*p* = 0.28) (Table 4).

**Table 4:** Adjusted associations of the number of teeth with ≥ 4 mm deep periodontal pockets and the number of bleeding sextants with arterial stiffening (Data set I, effective n = 153)

	β-estimate	95% CI	*p*-value
*Number of teeth with* ≥ *4 mm deep periodontal pockets*			
Peterson’s elastic modulus (mmHg)	15.80	− 3.99 to 35.58	0.12
Young’s elastic modulus (mmHg)	61.02	− 37.90 to 159.95	0.22
Carotid artery compliance (%/10 mmHg)	0.00	− 0.02 to 0.01	0.85
Beta stiffness index	0.01	0.01 to 0.02	0.46
*Number of bleeding sextants*			
Peterson’s elastic modulus (mmHg)	31.06	− 13.33 to 75.45	0.17
Young’s elastic modulus (mmHg)	121.16	− 100.63 to 342.96	0.28
Carotid artery compliance (%/10 mmHg)	− 0.02	− 0.05 to 0.02	0.37
Beta stiffness index	0.02	− 0.01 to 0.05	0.22

Adjusted β-estimates, their 95% confidence intervals (CI) and *p*-values

Data set I comprised never-smoking, non-diabetic, non-obese, non-rheumatic individuals without hypertension or coronary artery disease or ongoing lipid lowering medications. Adjusted for age, gender, educational level, serum lipid composition (triglycerides, LDL-cholesterol, HDL-cholesterol), BMI, level of physical activity, alcohol consumption and systolic blood pressure

### Data set II

The results in the unrestricted population in this data set were in line with the results in Data set I, with the exception that the adjusted β-estimates for the associations between Peterson’s elastic modulus and Young’s elastic modulus and the number of teeth with ≥ 4 mm deep periodontal pockets and the number of bleeding sextants were closer to null than in Data set I (Table 5).

**Table 5:** Adjusted associations of the number of teeth with ≥ 4 mm deep periodontal pockets and the number of bleeding sextants with arterial stiffening (Data set II, effective n = 527 and n = 526, respectively)

	β-estimate	95% CI	*p*-value
*Number of teeth with* ≥ *4 mm deep periodontal pockets*			
Peterson’s elastic modulus (mmHg)	3.40	− 5.59 to 12.38	0.46
Young’s elastic modulus (mmHg)	12.60	− 33.54 to 58.73	0.59
Carotid artery compliance (%/10 mmHg)	− 0.00	− 0.01 to 0.01	0.71
Beta stiffness index	0.00	0.00 to 0.01	0.62
*Number of bleeding sextants*			
Peterson’s elastic modulus (mmHg)	10.68	− 13.90 to 35.25	0.39
Young’s elastic modulus (mmHg)	74.65	− 51.43 to 200.73	0.25
Carotid artery compliance (%/10 mmHg)	− 0.01	− 0.03 to 0.01	0.46
Beta stiffness index	0.01	− 0.01 to 0.03	0.42

Adjusted β-estimates, their 95% confidence intervals (CI) and *p*-values

Data set II consisted dentate individuals aged 45–74. Adjusted for age, gender, educational level, serum lipid composition (triglycerides, LDL-cholesterol, HDL-cholesterol), BMI, level of physical activity, alcohol consumption, systolic blood pressure, smoking history, diabetes, coronary artery disease and rheumatoid arthritis

## Discussion

The aim in this population-based study among the Finnish adults aged 45–74 was to investigate whether an association exists between periodontal condition and arterial stiffness. Periodontitis has been associated with pathologies in arterial walls, such as impaired endothelial function and increased intima media thickness. The proposed biologic mechanisms behind these include local inflammatory effects by periodontal pathogens in arterial walls and periodontitis-induced low-grade systemic inflammation. As inflammation seems to play a role also in arterial stiffening, an association between periodontal inflammation and arterial stiffness could be expected. However, contrary to many earlier studies, the results of this study did not support the findings of earlier studies that have shown increased arterial stiffness along with an increased extent/severity of periodontitis.

Conflicting results of the association between periodontal disease and arterial stiffness have been published so far. The recent systematic review and meta-analysis by Darnaud and co-workers [16] summarized the results of 17 clinical studies of the association between periodontitis and arterial stiffness measured by pulse wave velocity (PWV). While five of the twelve cross-sectional studies found a significant association between arterial stiffness and periodontitis, four others did not show such an association. In three studies, a significant difference in arterial stiffness between the severe periodontitis group and the non-severe periodontitis group was observed, but the difference attenuated or became non-significant after adjusting for confounding factors such as age, gender, diabetes, smoking and systolic blood pressure. The results of the interventional studies, two of which were randomized clinical trials (RCTs), were inconclusive as to the reduction in PWV after periodontal therapy. Despite the conflicting results and the heterogeneity between the studies, the overall conclusion by the authors was that ‘patients with severe periodontitis have higher PWV compared to patients with non-severe periodontitis’. Of note is that the reviewed studies were conducted in specific populations, and many of them in individuals with systemic co-morbidities, which most likely resulted in biases.

Contrary to the findings of the present study, two earlier population-based studies reported an association of periodontal disease with arterial stiffness. In a Japanese study [13] among ≥ 40-year-old individuals, a linear dose-dependent association between mean periodontal pocket depth and arterial stiffness measured using carotid-ankle vascular index (CAVI) was observed. Another Japanese study [15] among community dwelling ≥ 75-year-old individuals reported that a higher degree of periodontal inflammation, measured as periodontal inflamed surface area, was linked with higher CAVI scores. In addition, individuals with severe periodontitis had significantly increased odds (ORs) for arterial stiffness when compared to individuals without severe periodontitis. Furthermore, supporting the results of the above studies, the severity and extent of periodontitis based on the mean periodontal attachment level and the percentage of sites with attachment level ≥ 5 mm appeared to be associated with arterial stiffness (CAVI) in a large 44–78-year-old Thai cohort [14].

One possible explanation for the contradictory results of the present study in relation to the results of the above studies may be a less severe periodontal situation. While the mean number of teeth with ≥ 4 mm deep periodontal pockets was only 3.8 in Data set I (Table 1) and 4.9 in the unrestricted Data set II (Table 2) of the present study, a total of 28% of the participants in the latter Japanese study [15] had severe periodontitis and 68% and 18% of the Thai subjects [14] had local and generalized periodontitis, respectively. Another explanation for the lack of association between the periodontal condition and arterial stiffness could be related to the robustness of the periodontal measures used; periodontal pocket depth was recorded at a tooth level and gingival bleeding at a sextant level. This robustness has likely caused measurement inaccuracy attenuating the strength of the studied associations.

In the present study, we made several restrictions to improve the homogeneity of the study population (Data set I). The restrictions reduced confounding related to obesity and eliminated the effects of smoking, diabetes, rheumatoid arthritis, hypertension, coronary artery disease and lipid-lowering medications, and therefore increased the credibility of the findings. Limiting the age of the study population to 45–64 years in Data set I was done to reduce age-related confounding. A disadvantage of these restrictions was that the study population became fairly small, which can be seen as wide confidence intervals and large *p*-values.

We also carried out analyses in a larger and unrestricted study population (Data set II). An essential question is, in which results do we trust: a smaller sample size, but higher validity due to more thorough control of the most important risks (hypertension, smoking, diabetes, cardiovascular diseases), or a larger and more heterogeneous sample where potential confounders are commonly controlled for using multivariate models (as for example in studies by Iwasaki and co-workers and Chansawang and co-workers). It may be worth emphasizing that in Data set I, the effect sizes (β-estimates) of Peterson’s elastic modulus and Young’s elastic modulus of the number of teeth with ≥ 4 mm deep periodontal pockets and the number of bleeding sextants were greater compared with the estimates obtained from Data set II. We interpreted this to mean that the association between periodontal parameters and arterial stiffness is confounded by extraneous factors, not fully controllable using multivariate models and, therefore, the associations should be studied in populations with as few competing risk factors for arterial stiffness as possible.

In terms of population selection, one strength in our study was that we had a relatively unselected population. It appeared that the data on periodontal pocketing and arterial stiffness in the unrestricted Data set II (Table 2) were quite similar to the respective data in the Health 2000 Survey population [25, 26]. A natural consequence of the strict restrictions related to smoking and non-oral diseases in Data set I was a lower mean number of teeth with ≥ 4 mm deep periodontal pockets (3.8 vs. 4.9) and, of the three earlier reported stiffness measures, a lower level of Young’s elastic modulus (5159 vs. 7040). On the other hand, the blood pressure rates, for example, in Data sets I and II were close to the rates in the Health 2000 Survey population [27]. In all, we consider that population selection had no major impact on the present results.

## Conclusion

This population-based study did not provide evidence of an association between periodontal condition and arterial stiffness.

## Data Availability

The data that support the findings of this study are available from the Finnish Institute for Health and Welfare (THL) but restrictions apply to the availability of these data, which were used under licence for the current study, and are thus not publicly available.

## Abbreviations

LDL-cholesterol: Low-density lipoprotein cholesterol
HDL-cholesterol: High-density lipoprotein cholesterol
BMI: Body mass index
CAC: Carotid artery compliance
YEM: Young’s elastic modulus
BSI: Beta stiffness index
PEM: Peterson’s elastic modulus
IMT: Intima media thickness
D_s_: Systolic diameter
D_d_: Diastolic diameter
P_s_: Systolic blood pressure
P_d_: Diastolic blood pressure
IPAQ: International Physical Activity Questionnaire
CI: Confidence interval
CAVI: Carotid-ankle vascular index
OR: Odds ratio
PWV: Pulse wave velocity
RCT: Randomized clinical trial

## References

[1] McEniery CM, Mäki-Petäjä KM, McDonnell BJ, Munnery M, Hickson SS, Franklin SS, Cockcroft JR, Wilkinson IB (2010). The impact of cardiovascular risk factors on aortic stiffness and wave reflections depends on age: the Anglo-Cardiff Collaborative Trial (ACCT III). Hypertension.

[2] AlGhatrif M, Strait JB, Morrell CH, Canepa M, Wright J, Elango P, Scuteri A, Najjar SS, Ferrucci L, Lakatta EG (2013). Longitudinal trajectories of arterial stiffness and the role of blood pressure: the Baltimore Longitudinal Study of Aging. Hypertension.

[3] Palombo C, Kozakova M (2016). Arterial stiffness, atherosclerosis and cardiovascular risk: pathophysiologic mechanisms and emerging clinical indications. Vasc Pharmacol.

[4] Cecelja M, Chowienczyk P (2009). Dissociation of aortic pulse wave velocity with risk factors for cardiovascular disease other than hypertension: a systematic review. Hypertension.

[5] Kaess BM, Rong J, Larson MG, Hamburg NM, Vita JA, Levy D (2012). Aortic stiffness, blood pressure progression, and incident hypertension. J Am Med Assoc.

[6] Ohkuma T, Ninomiya T, Tomiyama H, Kario K, Hoshide S, Kita Y, Inoguchi T, Maeda Y, Kohara K, Tabara Y, Nakamura M, Ohkubo T, Watada H, Munakata M, Ohishi M, Ito N, Nakamura M, Shoji T, Vlachopoulos C, Yamashina A (2017). Brachial-ankle pulse wave velocity and the risk prediction of cardiovascular disease. An individual participant data meta-analysis. Hypertension.

[7] Sanz M, del Castillo AM, Jepsen S, Gonzales-Juanatey JR, DÁiuto F, Bouchard P, Chapple I, Dietrich T, Gotsman I, Graziani F, Herrera D, Loos B, Madianos P, Michel JB, Perel P, Pieske B, Shapira L, Shechter M, Tonetti M, Vlachopoulos C, Wimmer G (2020). Periodontitis and cardiovascular diseases: consensus report. J Clin Periodontol.

[8] Paraskevas S, Huizinga JD, Loos BG (2008). A systematic review and meta-analyses on C-reactive protein in relation to periodontitis. J Clin Periodontol.

[9] Demmer RT, Trinquart L, Zuk A, Fu BC, Blomkvist J, Michalowicz BC, Ravaud P, Desvarieux M (2013). The influence of anti-infective periodontal treatment on C-reactive protein: a systematic review and meta-analysis of randomized controlled trials. PLoS ONE.

[10] Reyes L, Herrera D, Kozarov E, Roldá S, Progulske-Fox A (2013). Periodontal bacterial invasion and infection: contribution to atherosclerotic pathology. J Clin Periodontol.

[11] Schenkein HA, Loos BG (2013). Inflammatory mechanisms linking periodontal diseases to cardiovascular diseases. J Clin Periodontol.

[12] Mozos I, Malainer C, Horbanczuk J, Gug C, Stoian D, Luca CT, Atanasov AG (2017). Inflammatory markers for arterial stiffness in cardiovascular diseases. Front Immunol.

[13] Hayashida H, Saito T, Kawasaki K, Kitamura M, Furugen R, Iwasaki T, Hayashida Y, Nakazato M, Sekita T, Takamura N, Maeda T (2013). Association of periodontitis with carotid artery intima-media thickness and arterial stiffness in community-dwelling people in Japan: the Nagasaki Islands study. Atheroscler.

[14] Chansawang K, Lertpimonchai A, Siripaiboonpon N, Thienpramuk L, Vathesatogkit P, Limpijankit T, Charatkulangkun O (2021). The severity and extent of periosontitis is associated with cardio-ankle vascular index, a novel stiffness parameter. Clin Oral Investig.

[15] Iwasaki M, Kimura Y, Yamaga T, Yamamoto N, Ishikawa M, Wada T, Sakamoto R, Ishimoto Y, Fujisawa M, Okumiya K, Otsuka K, Matsubayashi K, Ogawa H (2021). A population-based cross-sectional study of the association between periodontitis and arterial stiffness among the older Japanese population. J Periodont Res.

[16] Darnaud C, Courtet A, Schmitt A, Boutouyrie P, Bouchard P, Carra MC (2021). Association between periodontitis and pulse wave velocity; a systematic review and meta-analysis. Clin Oral Investig.

[17] Kharazmi E, Moilanen L, Fallah M, Kaaja R, Kattainen A, Kähönen M, Jula A, Kesäniemi A, Luoto R (2007). Reproductive history and carotid intima-media thickness. Acta Obstet Gynecol.

[18] Salomaa V, Riley W, Kark JD, Nardo C, Folsom AR (1995). Non-insulin dependent diabetes mellitus and fasting glucose and insulin concentrations are associated with arterial stiffness indexes: the ARIC study: Atherosclerotic Risk in Communities Study. Circul.

[19] Heistaro S, editors. Methodology Report. Health 2000 Survey. Helsinki: Publications of the National Public Health Institute 2008; B26/2008.

[20] O’Rourke MF, Staessen JA, Vlachopoulos C, Duprez D, Plante GE (2002). Clinical applications of arterial stiffness; definitions and reference values. Am J Hypertens.

[21] Hirai T, Ohishi H, Honda N, Uchida H (1989). Ultrasonography of the liver. Rinsho Hoshasen.

[22] Vehkalahti M, Knuuttila M, Hausen H. Quality assurance of clinical examinations. In: Suominen-Taipale L, Nordblad A, Vehkalahti M, editors. Oral Health in the Finnish Adult Population, Health 2000 Survey. Helsinki: Publications of the National Public Health Institute 2004; B16/2004. p. 24–32.

[23] Leiviskä J, Salminen I, Sundvall J. Laboratory. In: Heistaro S, editors. Methodology report. Health 2000 Survey. Helsinki: Publications of the National Public Health Institute 2008; B26/2008. p. 76–8.

[24] Aromaa A, Koskinen S, editors. Health and functional capacity in Finland. Baseline results of the Health 2000 Health Examination Survey. Helsinki: Publications of the National Public Health Institute 2004; B12/2004.

[25] Knuuttila M, Suominen-Taipale L. Periodontal status, Oral Health in the Finnish adult population. Health 2000 Survey, Helsinki: Publications of the National Public Health Institute 2008. B25; 2005. p. 49–53.

[26] Koivistoinen T, Kööbi T, Moilanen L, Jula A, Lehtimäki T, Hyttinen J, Kähönen M (2011). Arterial tension time reflects subclinical atherosclerosis, arterial stiffness and stroke volume. Clin Physiol Funct Imaging.

[27] Niiranen TJ, Jula AM, Kantola IM, Reunanen A (2006). Comparison of agreement between clinic and home-measured blood pressure in the Finnish adult population: the Finn-HOME study. J Hypertens.

